# Explaining the travelling behaviour of migrants using Facebook audience estimates

**DOI:** 10.1371/journal.pone.0238947

**Published:** 2020-09-11

**Authors:** Spyridon Spyratos, Michele Vespe, Fabrizio Natale, Stefano Maria Iacus, Carlos Santamaria

**Affiliations:** European Commission, Joint Research Centre (JRC), Ispra, Italy; Beihang University, CHINA

## Abstract

The paper explores the travelling behaviour of migrant groups using Facebook audience estimates. Reduced geographical mobility is associated with increased risk of social exclusion and reduced socio-economic and psychological well-being. Facebook audience estimates are timely, openly available and cover most of the countries in the world. Facebook classifies its users based on multiple attributes such as the country of their previous residence, and whether they are frequent travellers. Using these data, we modelled the travelling behaviour of Facebook users grouped by countries of previous and current residence, gender and age. We found strong indications that the frequency of travelling is lower for Facebook users migrating from low-income countries and for women migrating from or living in countries with high gender inequality. Such mobility inequalities impede the smooth integration of migrants from low-income countries to new destinations and their well-being. Moreover, the reduced mobility of women who have lived or currently live in countries with conservative gender norms capture another aspect of the integration which is referring to socio-cultural norms and gender inequality. However, to provide more solid evidence on whether our findings are also valid for the general population, collaboration with Facebook is required to better understand how the data is being produced and pre-processed.

## Introduction

This article aims to study and explain the travelling behaviour of migrant groups at a global level using Facebook audience estimates. The main idea behind studying the geographical mobility of migrants groups is that by understanding their travelling behavior it is possible to have an indirect measure of migrant’s integration and well-being. Several studies associate reduced geographical mobility with increased risk of social exclusion [1], reduced psychological well-being [2] and lower-income [3, 4]. De Vos et al. [5] suggests that travel behaviour affects well-being through experiences during a) destination-oriented travel; b) activity participation enabled by travel; c) activities during destination-oriented travel; d) trips where travel is the activity; and e) through potential travel which is defined by Kaufmann, Bergman, & Joye [6] as motility.

There is limited research focusing on the travelling behaviour of migrant communities. Shin [7] study demonstrated that the possibilities and potential for movement are often limited for women and minorities. Other studies [8, 9] demonstrated that the Russian speakers in Estonia are less spatially mobile within Estonia, but more mobile regarding travels outside Estonia compared to the Estonian speakers. According to Georggi and Pendyala [3] African Americans and Hispanics in the US have reduced long-distance travel behaviour compared to the white population.

Several studies relied on non-traditional data such as mobile position data, air traffic data, Twitter data and IP address data to study human mobility. Masso, Silm, & Ahas [9] studied domestic and international spatial mobility by age, gender and language (Estonian or Russian) using passive mobile positioning data from an Estonian mobile phone operator. Silm, Ahas, & Nuga [10] studied gender mobility difference using both mobile position data and questionnaire survey in Estonia. Hawelka et al. [11] used Twitter data to estimate the volume of international travellers by country of residence. Fiorio et al., [12] used Twitter data to study short-term mobility and long-term migration within the US. Gabrielli, Deutschmann, Natale, Recchi, & Vespe, [13] used monthly air passenger traffic to study types of mobility and mobility trends at a global level. Finally, State, Ingmar, & Zagheni, [14] study inter-national mobility using IP data recorded from the logins of an initial sample of over 100 million users of Yahoo Web service.

Most of these studies are either limited to specific groups of migrants, to specific countries, or they are capturing traffic flows between countries. Still, a systematic and comparative analysis of migrants’ mobility behaviour by country of origin and destination at a global level is missing. When it comes to statistical data sources, to the best of our knowledge, there are no available data sets at a global level about the domestic or international travelling behaviour which target specifically migrants. In the UK, the International Passenger Survey (IPS) [15] collects information about passengers departing from and arriving in the UK by nationality and residence among other attributes. At the European level, Eurostat [16] provides on annual basis statistics about domestic and outbound touristic trips of EU residents but also in this case the data are not designed to represent specifically migrants’ populations in each Member State.

Data from Facebook can help to address the absence of statistics about the mobility of migrant groups at a global level. Facebook, despite its limitations, offers unprecedented possibilities to capture new insights on the sociodemographic and behavioural characteristics of the migrants’ population broken down by country of residence, country of origin, age and gender. To the best of our knowledge, this is the first study to explore the travelling attribute of the Facebook Advertising platform [17]. By considering this additional attribute, we build on past research that makes use of data from Facebook to study the size of migrant stocks [18, 19], migrant assimilation [20] and gender inequalities [21]. The use of non-traditional data sources, like Facebook, can potentially complement traditional data as a source for statistics.

This article is organised as follows. The data section presents the Facebook audience estimates used in this study and includes some simple correlations to test the association between the travelling behaviour of Facebook users and travel data available from the UK International passenger survey. The methodology section describes six models used to explain how the travelling behaviour of Facebook users is affected by gender, age and countries of the previous residence of the migrants. In the results and discussion section, we present the outcome of the Facebook data analysis, and the conclusions are outlined in the final section.

## Data

The Facebook Advertising platform, allows users to design targeted advertisements on the Facebook family of applications, by selecting the characteristics of the targeted audience. These characteristics of the Facebook users include, for example, age, gender, location, country of previous residence and, particularly relevant for this study, whether they are “frequent travellers” or “frequent international travellers”. Once users have selected the characteristics of the Facebook population that they wish to target with the advertisement campaign, the advertising platform provides an estimate of the number of daily active users (DAU) and monthly active users (MAU) that fulfil these characteristics. We collected these estimates to generate aggregate estimates on the share of frequent travellers and frequent international travellers in the total population, by country of residence, age, and gender. We similarly collect the same estimates for the population that Facebook classifies as having lived abroad for all pairs of countries of previous and current destination as well as for the Facebook users who have not lived abroad.

Facebook classifies its users as “frequent international travellers” based on whether they have travelled abroad more than once in the past six months [17]. Since the data collection phase of this study took place from September 2019 to October 2019, it is expected that Facebook captured for the classification of the Facebook users as “frequent international travellers” international trips made up to six months before the date of data collection, meaning from March/April 2019 to September/October 2019. Facebook classifies its users as “frequent travellers” based on whether their activities on Facebook suggest that they are frequent travellers [17]. For the “frequent travellers” attribute Facebook does not provide any reference neither about the time period of the travel nor about the minimum distance of the travel required to classify its users as “frequent travellers”. The definition of frequent traveller is very generic since Facebook does not provide details on how it classifies a user as a “frequent traveller”. Thus we don’t know the destination of the travel, the purpose of the travel or whether the travels refer to short, middle or long-distance mobility. We decided to use the “frequent traveller” attribute in our analysis since we perform a comparative analysis of the same attribute between different “migrant” groups and the “non-migrant” population. The classification of Facebook users as “frequent international travellers” and “frequent travellers” is not likely to be self-reported. This is because, as of September 2019, the 51% of Facebook users who were mainly accessing Facebook through a mobile device were classified as frequent travellers, while only the 2.5% of those who were not primarily accessing Facebook using a mobile device were classified as frequent travellers. We can thus assume that Facebook is using the location of the mobile devices to classify users as frequent travellers or not.

To represent migrants, we rely on the classification of Facebook users as having “lived in country X”, which is based on whether they used to live in country X and they now live abroad. This classification is provided for the 89 countries of previous residence listed in S1 Table in Annex A. The key criteria that Facebook uses for identifying the previous residence of a user is the “hometown”, “current city”, and “other places lived”, as well the network structure of Facebook friendships [19]. In this study, we use the term Facebook “migrants” to describe Facebook users who have been classified as having lived in a country other than the country of their current residence and the term Facebook “non-migrants” to describe the users who have not lived in any other country than the country of their current residence.

To collect Facebook audience estimates, we have developed a python script, which was used to query the Facebook Marketing Application Programming Interface (API) [22] and store the data to a Postgresql database. Using this python script, we collected for each age group *a* ∈ *[15–24, 25–34, 35–44, 45–54, 55–64, 15–64]*, gender *g*∈ *[Male*, *Female*, *Both]*, country of current residence *c*, and country of previous residence *p* ∈ *[countries in S1 Table]*, as well as, for non-migrants *n* and total Facebook users *t*, the number of Facebook MAU *fb*_*a*,*g*,*c*,*p/n/t*_; the number of Facebook MAU who are classified as “frequent international travellers” *fit*_*a*,*g*,*c*,*p/n/t*_; and the number of Facebook MAU who are classified as “frequent travellers” *ft*_*a*,*g*,*c*,*p*_. We restricted the analysis only to Facebook users who primarily access Facebook using mobile devices since, as we explained earlier in this section, access from mobile devices represent a key feature for Facebook to classify the travelling behaviour of users. Due to the high number of variables collected and the API rate limits of approximately one API call every 10 seconds, the data collection period spanned from 4 September 2019 to 30 October 2019.

A first limitation of the collected Facebook audience estimates is that values are returned with a minimum threshold of 1000 “confidentiality threshold”. For example, if a selected group have 10 MAU, the Facebook estimate will be 1000 MAU. As a result, in this study, we are only able to use estimates about demographic groups with higher than 1000 MAU. A second limitation is that Facebook’s Marketing API only provides a rounded estimate of MAU. The applied rounding is proportional to the number of MAU, for example, for MAU values between 1000 and 10,000, the rounding precision is 100; for values between 10,000 and 100,000, the rounding precision is 1000; and so forth.

**Fig 1:**
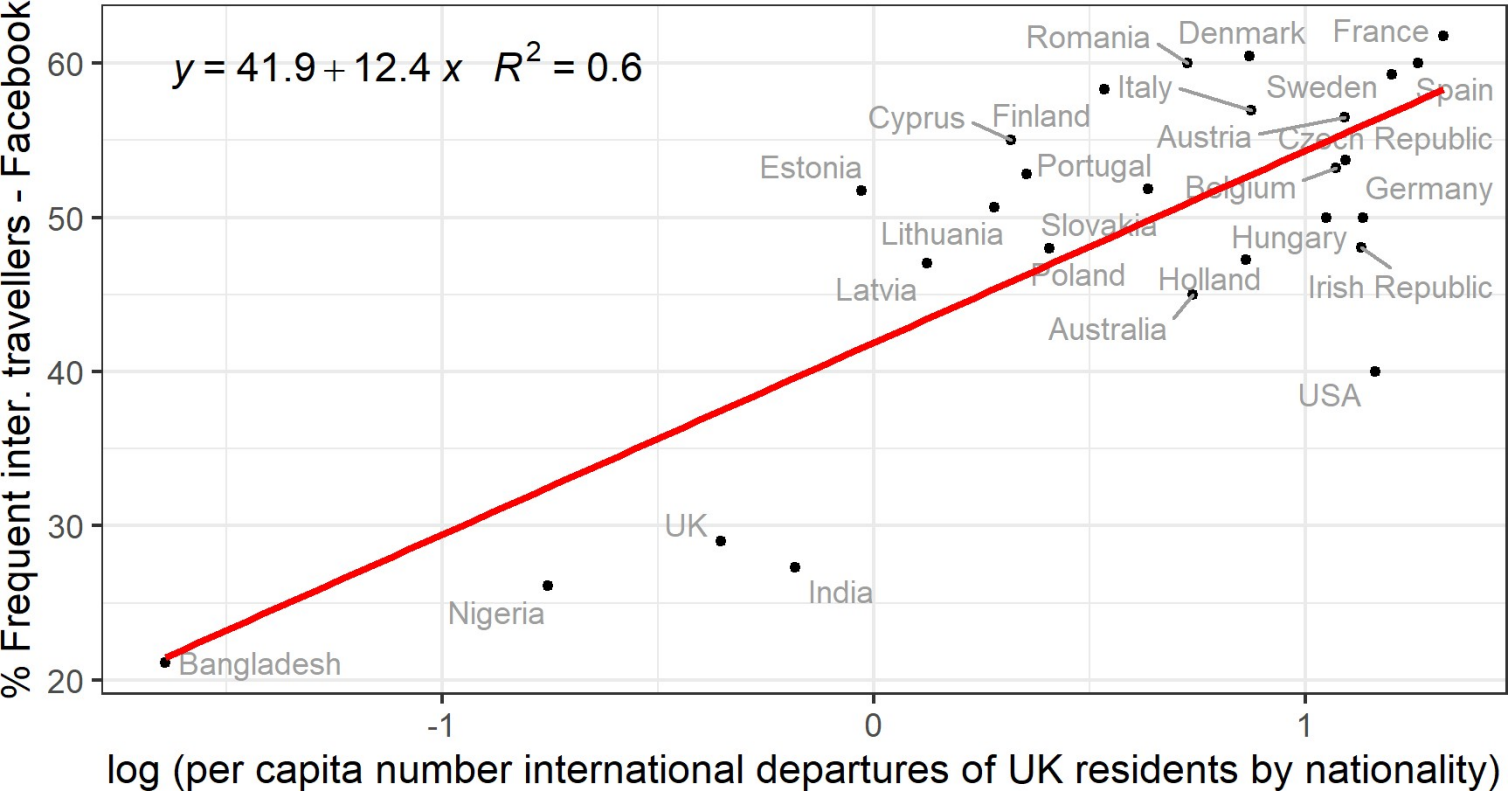
International travelling behaviour in the UK by nationality/previous residence. The x-axis shows the log of the per capita number of international departures of UK residents by nationality from March 2018 to August 2018 as estimated by the UK IPS survey. The y-axis shows the percentage of Facebook users who live in the UK and have made at least one international travel from March 2019 to August 2019 by country of previous residence.

To assess the reliability of Facebook derived travelling estimates, we compared them with relevant statistics regarding international travels of UK residents. The International Passenger Survey (IPS) [15] collects information about passengers departing from and arriving in the UK by nationality and residence, among other attributes. Fig 1 shows the comparison between the log of the per capita number of international departures of UK residents by nationality during the time period March to August 2018 and the percentage of Facebook users who live in the UK and have at least made one international travel during the time period March to August 2019 by country of previous residence. To estimate the per capita number of international departure of UK residents by nationality, we divided the estimated number of international departures by the stock of UK migrants by citizenship available from Eurostat [22] for 2018. There is a good correlation between the two variables compared *(R*^*2*^ *= 0*.*6*, *p<0*.*001)*, even though they differ in terms of reference time and definitions used to measure both international travelling behaviour and country of birth or previous residence. The high *R*^*2*^ is mainly due to countries with low values in both the x and y axes. Given the aim of this study, the below correlation for the case of UK shows that Facebook data can be used to identify, with a good degree of approximation, migrant groups with reduced international travelling behaviour such as Bangladeshi migrants.

**Fig 2:**
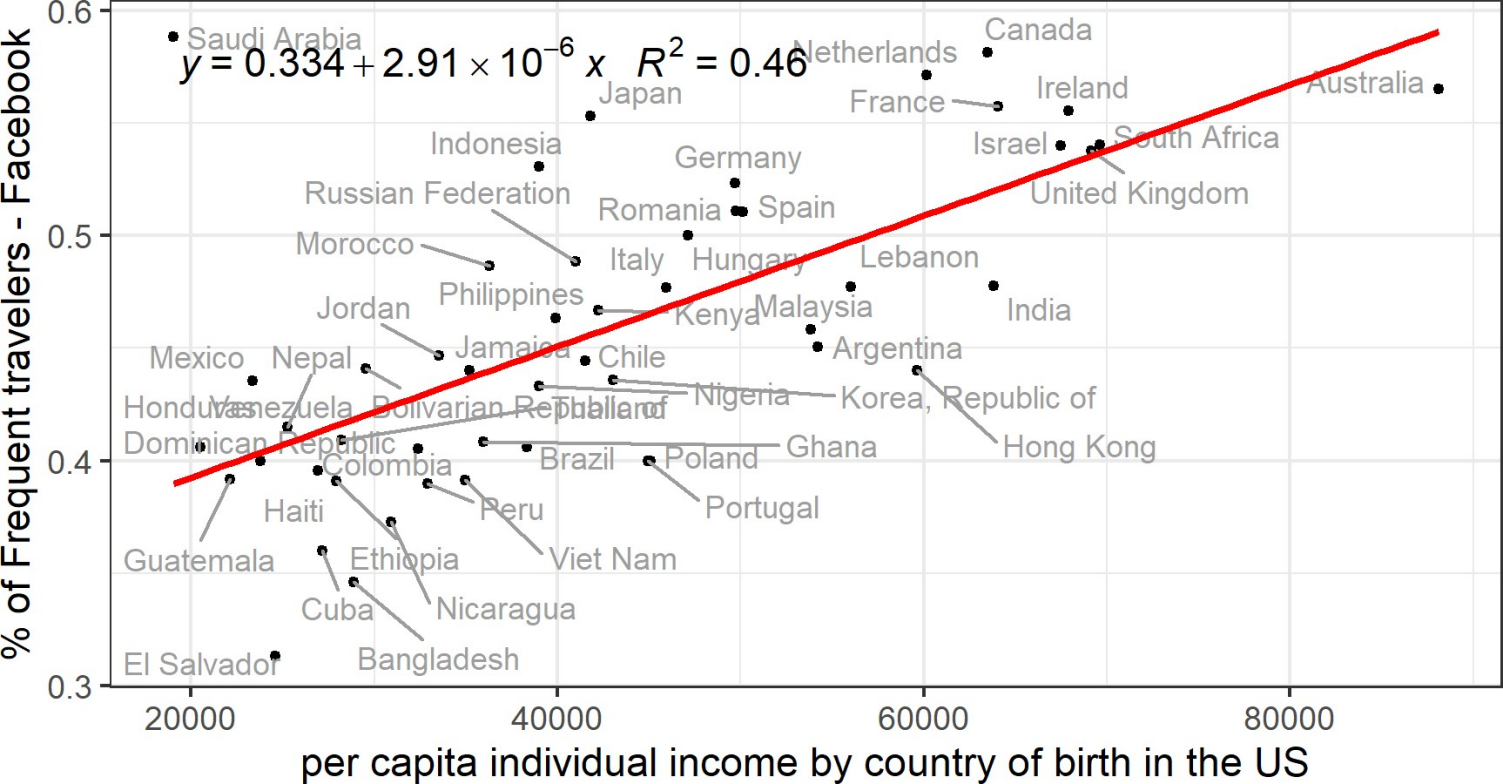
Correlation between income and travelling behaviour. The x-axis shows the per capita individual income of US residents by country of birth, and the y-axis shows the percentage of Facebook users who are frequent travellers by country of previous residence in the US.

A similar analysis has been carried out for the US. In this case, to the absence of national and international travelling statistics by country of origin, we decided to compare the frequent travelling Facebook attribute with income statistics. The income and the travelling frequency attribute do not measure the same phenomenon but the income explains part of the travelling behaviour. As Fig 2 shows, the estimated per capita annual income of individuals in US dollars for 2017 by country of birth in the US [23] is correlated *(R*^*2*^ *= 0*.*46*, *p<0*.*001)* with the percentage of frequent travellers in the US by country of previous residence.

## Methodology

All data from Facebook’s Marketing API were provided to us in a fully anonymised, aggregated and rounded format with a confidentiality threshold of 1000 or more users. Thus this data can be considered to be ‘statistical data’ and not ‘personal data’. The mobility of Facebook users is expected to be affected by their demographic characteristics, such as age and gender and by the characteristics of the countries of their current residence and, in case these users are migrants, the characteristics of the country of the previous residence. We use six regression models to test the role of these variables in explaining the travelling behaviour of Facebook users considering both migrant and the non-migrant populations. The models *a* and *b* presented below explain the mobility of Facebook non-migrant users while the models *c*, *d*, *e*, and *f* refer to Facebook migrant users. The models *a*, *c*, and *e* explain the “frequent travellers” Facebook attribute while the models *b*, *d* and *f* explain the “frequent international travellers” attribute.

**Table:** 

***Model a*:** *Frequent travellers*, *Non-migrants*	*ft_per*_*a*,*g*,*c*,*n =*_ *B*_*0*_ *+ B*_*1*,*c*_ *Country*_*c*_ *+ B*_*4*,*a*_ *age*_*a*_ *+ B*_*5*_ *female + B*_*6*_ *(1-gdi*_*c*_*) B*_*10*_ *pen_rate*_*a*,*g*,*c*,*t*_ *+ ε*_*a*,*g*,*c*_
***Model b*:** *Frequent international travellers*, *Non-migrants*	*fιt_per*_*a*,*g*,*c*,*n =*_ *B*_*0*_ *+ B*_*1*,*c*_ *Country*_*c*_ *+ B*_*4*,*a*_ *age*_*a*_ *+ B*_*5*_ *female + B*_*6*_ *(1-gdi*_*c*_*) + B*_*10*_ *pen_rate*_*a*,*g*,*c*,*t*_ *+ ε*_*a*,*g*,*c*_
***Model c*:** *Frequent travellers*, *Migrants*	*ft_per*_*a*,*g*,*c*,*p =*_ *B*_*0*_*+ B*_*1*,*c*_ *Country*_*c*_ *+ B*_*4*,*a*_ *age*_*a*_*+ B*_*5*_ *female + B*_*6*_ *(1-gdi*_*c*_*) + B*_*7*_ *(1-gdi*_*p*_*) + B*_*8*_ *log(dist*_*c*,*p*_*) + B*_*9*_ *log(GDPpc*_*p*_*) + B*_*10*_ *pen_rate*_*a*,*g*,*c*,*t*_ *+ ε*_*a*,*g*,*c*,*p*_
***Model d*:** *Frequent international travellers*, *Migrants*	*fit_per*_*a*,*g*,*c*,*p =*_ *B*_*0*_*+ B*_*1*,*c*_ *Country*_*c*_ *+ B*_*4*,*a*_ *age*_*a*_ *+ B*_*5*_ *female + B*_*6*_ *(1-gdi*_*c*_*) + B*_*7*_ *(1-gdi*_*p*_*) + B*_*8*_ *log(dist*_*c*,*p*_*) + B*_*9*_ *log(GDPpc*_*p*_*) + B*_*10*_ *pen_rate*_*a*,*g*,*c*,*t*_ *+ ε*_*a*,*g*,*c*,*p*_
***Model e*:** *Frequent travellers*, *Migrants*, *no country fixed effect*	*ft_per*_*a*,*g*,*c*,*p =*_ *B*_*0*_ *+ B*_*2*_ *ft_per*_*a*,*g*,*c*,*n*_ *+ B*_*4*,*a*_ *age*_*a*_ *+ B*_*5*_ *female + B*_*7*_ *(1-gdi*_*p*_*) + B*_*8*_ *log(dist*_*c*,*p*_*) + B*_*9*_ *log(GDPpc*_*p*_*) + B*_*10*_ *pen_rate*_*a*,*g*,*c*,*t*_ *+ ε*_*a*,*g*,*c*,*p*_
***Model f*:** *Frequent international travellers*, *Migrants*, *no country fixed effect*	*fit_per*_*a*,*g*,*c*,*p =*_ *B*_*0*_ *+ B*_*3*_ *fit_per*_*a*,*g*,*c*,*n*_ *+ B*_*4*,*α*_ *age*_*a*_*+ B*_*5*_ *female + B*_*7*_ *(1-gdi*_*p*_*) + B*_*8*_ *log(dist*_*c*,*p*_*) + B*_*9*_ *log(GDPpc*_*p*_*)*_*+*_ *B*_*10*_ *pen_rate*_*a*,*g*,*c*,*t*_ *+ ε*_*a*,*g*,*c*,*p*_

As the dependent variables in the above-presented models, we used the percentages of frequent international travellers *fit_per*_*a*,*g*,*c*,*p/n*_ and frequent travellers *ft_per*_*a*,*g*,*c*,*p/n*_. These percentages are estimated using Eq (1) and Eq (2) by dividing the number of Facebook MAU who are classified as “Frequent international travellers” *fit*_*a*,*g*,*c*,*p/n*_ or “Frequent travellers” *ft*_*a*,*g*,*c*,*p/n*_ of age *a*, gender *g*, country of residence *c*, and of country of previous residence *p* or *n* of non-migrants by the number of Facebook MAU *fb*_*a*,*g*,*c*,*p/n*_ of age *a*, gender *g*, country of residence *c*, and of country of previous residence *p* or *n* of non-migrants.

$$fit\_per_{a,g,c,p/n}=fit_{a,g,c,p/n}/fb_{a,g,c,p/n}$$ (1)

$$ft\_per_{a,g,c,p/n}=ft_{a,g,c,p/n}/fb_{a,g,c,p/n}$$ (2)

In the models *a*, *b*, *c* and *d* we used the current-country specific fixed effects, which means that each country of residence has its coefficient, except the one country which is used as a reference. In the models *e* and *f* we used as independent variables the percentage of non-migrant Facebook users who are frequent travellers or frequent international travellers respectively by country of current residence, age and gender.

The per capita income in the country of the previous residence is expected to have a positive impact on the mobility of Facebook users. As income measure, we used the Gross Domestic Product (GDP) per capita *GDPpc* expressed in current United States (US) dollars available from the World Bank [24]. Gender inequality is another variable that could affect the travelling of female Facebook users. We used the Gender Development Index (GDI) available from the United Nations Development Programme [25]. The GDI reflects gender-based disparities in three dimensions, health, knowledge and living standards, and it is the ratio of female and male Human Development Index (HDI). GDI is equal to one when, women and men have the same HDI, above one when female fares better than male and below one in the opposite case. GDI is available for 164 countries. In our models for observations that describe male travelling behaviour, we fixed the *gdi*_*c*_ or the *gdi*_*p*_ values to 1.

The distance between the countries of current and previous residence of a Facebook user is expected to have an impact on the number of trips back to the country of previous residence. The trips of Facebook users to the country of previous residence accounts for a proportion of the total international travels since not all the international trips are towards the country of previous residence. In our models, we used the geodetic distances *dist*_*c*,*p*_ between countries of previous and current residence from the CEPII’s GeoDist dataset [26]. We selected to use the “dist” variable of the CEPII’s GeoDist dataset which describes the geodesic distances between the most important cities/agglomerations of each country in terms of population.

Statistical analyses based on non-randomly selected samples of the population, such as the groups of the population who use Facebook through a mobile device, can lead to erroneous conclusions. A possible solution for correcting the selection bias of Facebook users would be the use of the Heckman correction [27]. However, since we rely on Facebook audience estimates on aggregated form and not on individual-level data, the use of Heckman correction is not feasible. To overcome this limitation, we assumed that the smaller the proportion of users who access Facebook mainly through a mobile device to the real population is, the higher is the probability that this sample will represent the most tech-savvy and wealthy part of the population which is more likely to be a frequent traveller and a frequent international traveller. The only exception to this hypothesis is the age group 15–25 where low Facebook use may be due to the use of alternative social media applications such as Instagram.

To estimate the selection bias due to the use of statistics that refer to a population who uses Facebook through a mobile device, we introduce as a variable to our models the penetration rate of the Facebook usage, *pen_rate*_*a*,*g*,*c*,*t*_. The penetration rate is estimated using Eq (3) by dividing the total number of Facebook users who access Facebook mainly through a mobile device which includes both Facebook migrants and non-migrant users *fb*_*a*,*g*,*c*,*t*_ of age, gender and country of residence by the population *UNDESA_pop*_*a*,*g*,*c*_ of age, gender and country of residence taken from the UNDESA statistics for the year 2019 (medium projection variant) [28]. We assume that cases of lower penetration rates for users of age 15–24 in respect of the age group 25–34, are determined by the use of other social media rather than by differences in technology adoption or wealth, in these cases we assigned the penetration rate of the age group 25–34 to the age group 15–24.

$$pen\_rate_{a,g,c,t=}fb_{a,g,c,t}/UNDESA\_pop_{a,g,c}$$ (3)

We fitted the above described six regression models using the Ordinary Least Squares (OLS) method, as well as using the Adaptive Elastic Net (AdaENet) method [29]. For the AdaENet, we used 80% of the observations as training data and the remaining 20% as testing data, and a 50-fold cross-validation for selecting the optimal lambda penalization parameter. We used a fixed alpha parameter equal to 0.5 to perform an equal combination of Ridge and Lasso regression. We implemented the AdaENet method using the “glmnet” package of the R software [30].

The decision of pairing OLS with AdaENet is because the second allows for contextual model selection (the Lasso contribution) and shrinkage (the Ridge contribution) estimation. Lasso tends to produce parsimonious models (by dropping some of the coefficients) which perform very well in predictions, while Ridge allows keeping in the model correlated coefficients, and this is very good for explaining the impact of group of variables on the outcome without necessarily dropping some of the coefficients. When OLS and AdaENet agree on the sign and amplitude of the coefficients, it is a good confirmation of the quality of the model in terms of descriptive and predictive power. AdaENet coefficients are usually smaller than the corresponding OLS ones but standard errors cannot be easily obtained. On the other hand, OLS makes it possible to evaluate the significance of those coefficients. For this reason, we present both evidence in Table 1 of the next section.

**Table 1:** Regression models using the OLS method and the AdaENet method.

Model description		Model a: *Frequent travellers*, *Non-migrants*	Model b: *Frequent international travellers*, *Non-migrants*	Model c: *Frequent travellers*, *Migrants*	Model d: *Frequent international travellers*, *Migrants*	*Model e*: *Frequent travellers*, *Migrants*, *no country fixed effect*	*Model f*: *Frequent international travellers*, *Migrants*, *no country fixed effect*
dependent variables		*ft_per*_*a*,*g*,*c*,*n*_	*fit_per*_*a*,*g*,*c*,*n*_	*ft_per*_*a*,*g*,*c*,*p*_	*fit_per*_*a*,*g*,*c*,*p*_	*ft_per*_*a*,*g*,*c*,*p*_	*fit_per*_*a*,*g*,*c*,*p*_
Ordinary Least Squares	*B*_*1*,*c*,_ Country of residence dummies	127 out of 141 signifi. at p < 0.1	115 out of 141 signifi. at p < 0.1	92 out of 105 signi. at p < 0.1	63 out of 99 signifi. at p < 0.1		
	*B*_*2*,_ *ft_per*_*a*,*g*,*c*,*n*_					0.843***	
	*B*_*3*,_ *fit_per*_*a*,*g*,*c*,*n*_						0.769***
	*B*_*4*,*25–34*,_ age 25–34 (ref. 15–24)	-0.011***	-0.015***	-0.011***	-0.011**	-0.005.	0.007, p = 0.13
	*B*_*4*,*35–44*,_ age 35–44	-0.016***	-0.022***	-0.025***	-0.022***	-0.026***	-0.016***
	*B*_*4*,*45–54*,_ age 45–54	-0.033***	-0.037***	-0.046***	-0.052***	-0.047***	-0.052***
	*B*_*4*,*55–64*,_ age 55–64	-0.067***	-0.064***	-0.088***	-0.083***	-0.078***	-0.088***
	*B*_*5*,_ female (ref. male)	-0.018***	-0.016***	-0.011***	-0.008.	0.000, p = 0.77	-0.002, p = 0.58
	*B*_*6*,_ (1—gdi_*c*_)	-0.258***	-0.224***	-0.281***	-0.525***		
	*B*_*7*,_ (1—gdi_*p*_)			-0.208***	-0.254***	-0.235***	-0.302***
	*B*_*8*,_ log (*dist*_*c*,*p*_)			-0.017 ***	-0.030***	-0.026***	-0.040***
	*B*_*9*,_log (*GDPpc*_*p*_)			0.012***	0.016***	0.016***	0.020***
	*B*_*10*,_ *pen_rate*_*a*,*g*,*c*,*t*_	-0.021*	-0.030**	-0.039***	-0.056**	-0.075***	-0.170***
	*B*_*0*,_ Intercept	0.608***	0.235***	0.690***	0.551***	0.260***	0.412***
	Multiple R^2^	0.987	0.952	0.873	0.752	0.804	0.643
	degrees of freed.	1023	1014	5649	2937	5848	3100
Adaptive Elastic Net	*B*_*1*,*c*,_ Country of residence dummies	141 included out of 141	140 included out of 141	105 included out of 105	92 included out of 93		
	*B*_*2*,_ *ft_per*_*a*,*g*,*c*,*n*_					0.839	
	*B*_*3*,_ *fit_per*_*a*,*g*,*c*,*n*_						0.767
	*B*_*4*,*25–34*,_ age 25–34 (ref. 15–24)	-0.007	-0.013	-0.010	-0.008	-0.0034	0.006
	*B*_*4*,*35–44*,_ age 35–44	-0.012	-0.022	-0.023	-0.015	-0.023	-0.011
	*B*_*4*,*45–54*,_ age 45–54	-0.027	-0.035	-0.044	-0.050	-0.045	-0.048
	*B*_*4*,*55–64*,_ age 55–64	-0.060	-0.063	-0.084	-0.079	-0.071	-0.082
	*B*_*5*,_ female (ref. male)	-0.018	-0.017	-0.011	-0.008	0.0003	-0.002
	*B*_*6*,_ (1—gdi_*c*_)	-0.258	-0.230	-0.278	-0.533		
	*B*_*7*,_ (1—gdi_*p*_)			-0.211	-0.270	-0.226	-0.334
	*B*_*8*,_ log (*dist*_*c*,*p*_)			-0.017	-0.030	-0.026	-0.040
	*B*_*9*,_log (*GDPpc*_*p*_)			0.012	0.018	0.016	0.019
	*B*_*10*,_ *pen_rate*_*a*,*g*,*c*,*t*_	-0.016	-0.027	-0.029	-0.046	-0.0867	-0.160
	*B*_*0*,_ Intercept	0.471	0.211	0.738	0.48	0.2533	0.423
	Multiple R^2^	0.979	0.929	0.865	0.772	0.805	0.645
	No. of observation	1172	1163	5765	3047	5859	3111

p < 0.1

* p < 0.05

** p < 0.01

*** p < 0,001.

Before fitting our models, we filtered and cleaned the data that we used in the six proposed models. First, we took into consideration countries of residence with at least two available gender-age observations. Second, we did not take into consideration the age-gender-residence observations with Facebook low penetration rate pen_rate_a,g,c,t,_ less than 10%. This threshold is indented to exclude countries where Facebook is not the popular social media application like Russia and Uzbekistan as well as age-gender-residence observations with a low proportion of users who access Facebook mainly through a mobile device to the real population, to avoid bias linked to a very poor representation of Facebook in the overall population of the country. Finally, we excluded two countries out of 89 countries of previous residence listed in the S1 Table. China was excluded from the analysis since Facebook use is restricted in that country. Greece was also excluded as a country of previous residence since Facebook audience estimates for users who have lived in Greece are strongly underestimated most likely due to a Facebook classification error. As of December 2019, Facebook was reporting only 3,800 users who have lived in Greece and now live abroad while according to UNDESA [31] in 2017 there were 993,000 Greek-born citizens that live abroad.

## Results

**Fig 3:**
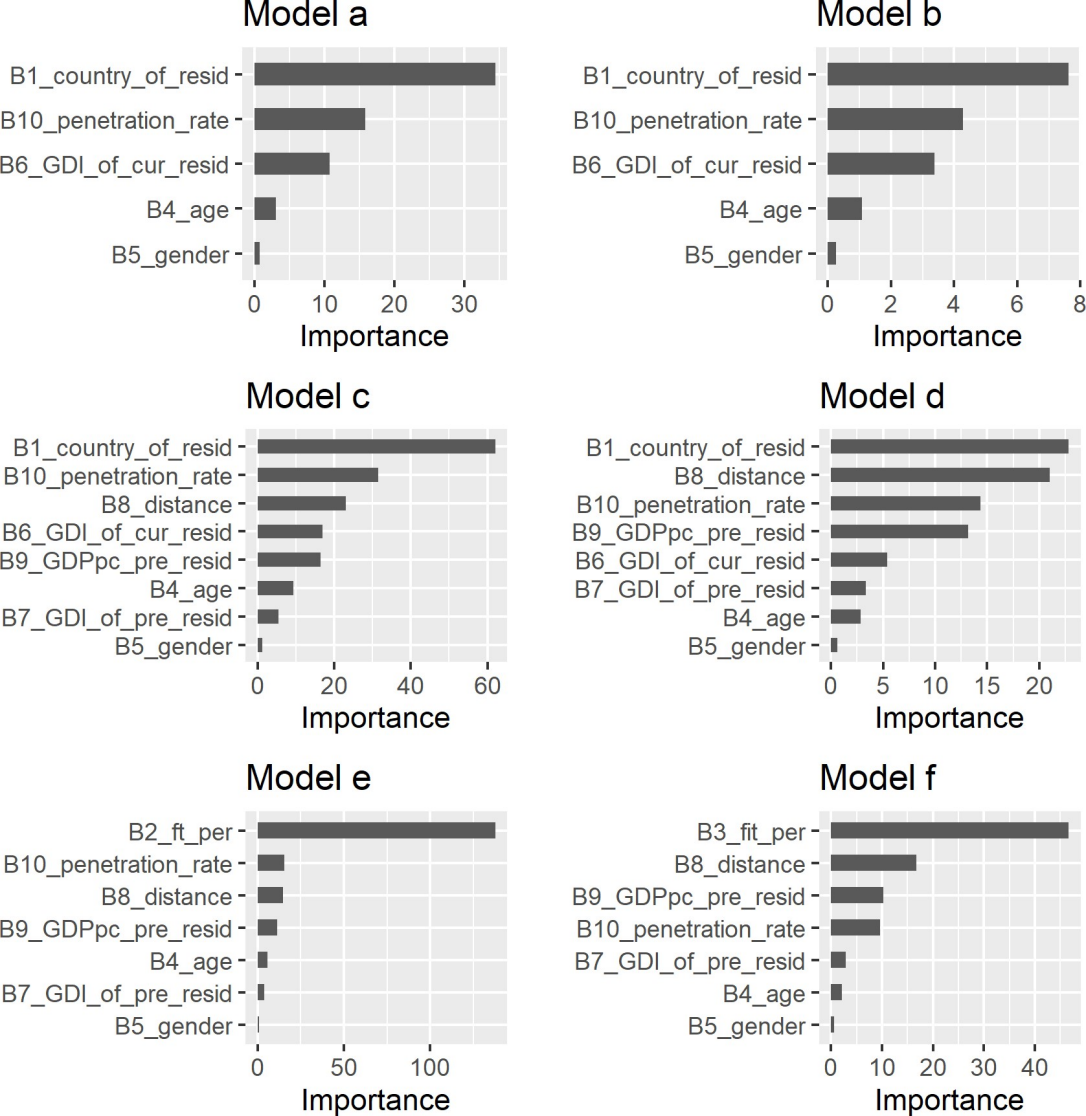
Variable importance for each independent variable using the Random Forest method.

The main contribution of this research is that we found strong indications that the frequency of travelling is lower for Facebook users migrating from low-income countries and for women migrating from countries with high gender inequality. In Table 1, we present the unstandardized coefficients as well as the accuracies of the proposed six models using the OLS method and the AdaENet method. In Fig 3 we present the importance of the variables which were used in each of the six models. The variable importance was estimated using the impurity (Gini) importance of the Random Forest classifier available in package “ranger” of the R software [32]. This importance measure summarizes how frequently a variable is determinant in predicting the outcome variable in a Random Forest.

In line with the literature [9, 10, 33, 34], the models’ unstandardized coefficients reported in Table 1 show that women travel slightly less than men and the elderly travel less than the young. As shown in Fig 3 the GDI plays a much more important role in explaining gender inequalities in travelling behaviour that the gender categorical variable. The GDI of both the country of previous and current residence *gdi*_*p*_ and *gdi*_*c*_ is correlated with the general as well as international travelling mobility of Facebook female users. This means that female Facebook users who have lived or currently live in countries with conservative gender norms are travelling less compared to female Facebook users who live or have lived in countries where both genders fare equally. This relation is pointing to the possibility of capturing through the analysis of mobility patterns another aspect of the integration of migrants which is pertaining to socio-cultural norms and gender inequality.

The per capita GDP of the country of previous residence of a Facebook migrant user *GDPpc*_*p*_ is positively correlated with general as well as international travelling mobility. This positive effect and the importance of this variable corroborates the main idea that mobility patterns may offer an indication of the wealth of migrants.

As shown in see Fig 3, the distance *dist*_*c*,*p*_ between the country of current and previous residence of a Facebook user, plays an important role in their travelling behaviour. The *B*_*9*,_ unstandardized coefficient in all the four models, is negative, and it is higher and more important in the models *d* and *f* that explicitly describe the international travelling behaviour of Facebook migrant users. This is because part of the international trips is expected to have as destination the country of the previous residence. The inclusion of this variable in the models is important to neutralise the impact of the cost of reaching the home-country on migrants mobility.

The Facebook penetration rate *pen_rate*_*a*,*g*,*c*,*t*_ is negatively correlated with the percentage of frequent or frequent international travellers in all the models. This variable is an important element in our model to compensate for the bias introduced by the over-representation in Facebook of the most tech-savvy and wealthy part of the population, which is also more likely to include frequent travellers and frequent international travellers.

**Fig 4:**
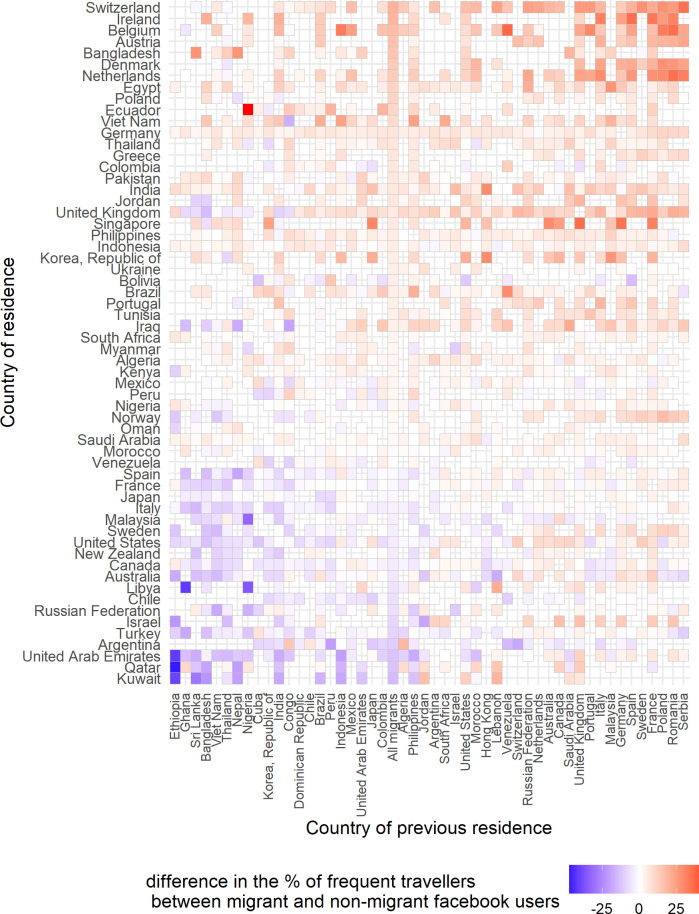
The heatmap of frequent travellers. The difference in the percentage of frequent travellers between Facebook migrant users and Facebook non-migrant users of age 15 to 64 for each country of residence. Empty cells mean no available data. The heatmap includes the 60 countries of residence with the highest number of migrant groups and the 50 countries of previous residence with the highest presence in the selected 60 countries of residence.

**Fig 5:**
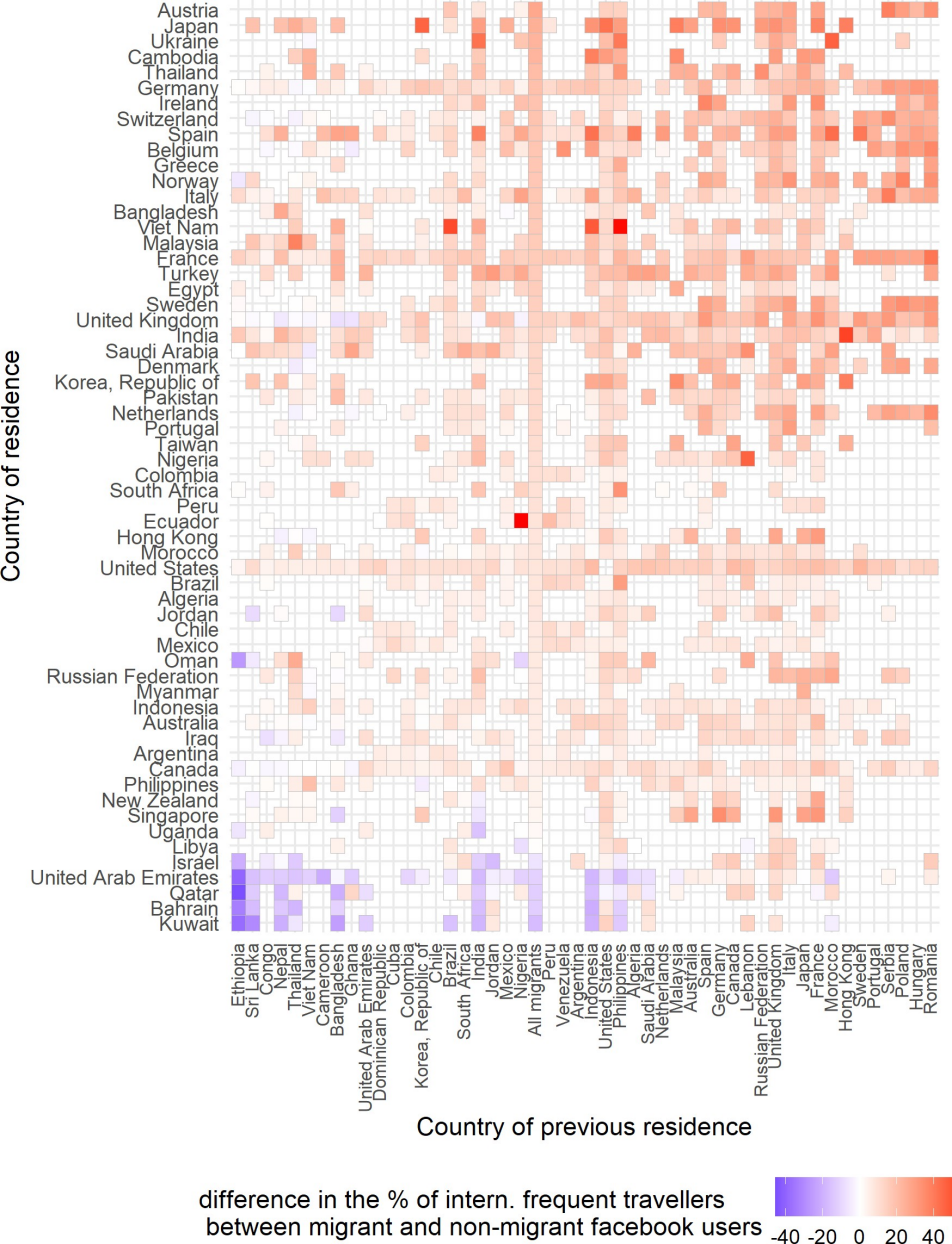
The heatmap of frequent international travellers. The difference in the percentages of frequent international travellers between Facebook migrant users and Facebook non-migrant users of age 15 to 64 for each country of residence. Empty cells mean no available data. The heatmap includes the 60 countries of residence with the highest number of migrant groups and the 50 countries of previous residence with the highest presence in the selected 60 countries of residence.

Finally, we provide a descriptive representation of the two Facebook attributes, namely the ‘frequent traveller’ in Fig 4 and of the ‘frequent international traveller’ in Fig 5. Apart from explaining the travelling behaviour of Facebook migrants based on the income and gender inequalities, we also identified migrant groups which according to Facebook audience estimates have very limited mobility, such as the Ethiopians and the Bangladeshis in Bahrain and Kuwait. This low mobility might represent domestic workers who are recruited throught the Kafala system. Kafala system is a government policy used to organise and control the migrant workers in the Gulf Cooperation Council countries [35]. Finally, we also find that Facebook users who have lived in east European countries and now live in west European countries are very mobile. However, we do not know to what percentage these Facebook users represent cross-border seasonal workers or permanent migrants.

## Discussion and conclusions

The main objective of this paper has been to examine the travelling behaviour of different migrant groups in multiple countries using Facebook audience estimates. Based on Facebook audience estimates we found strong indications that Facebook migrant users who have lived in low-income countries are less mobile than Facebook migrants from rich countries. We also found that female Facebook users who have lived or currently live in countries where gender inequality is high are less mobile than female Facebook users who have lived or live in more gender-equal countries. We were able to identify Facebook migrant users with reduced travelling behaviour, such as Facebook users who have lived in Ethiopia, and now live in the Gulf countries.

There are various limitations related to the use of Facebook audience estimates. A first limitation is that Facebook penetration rates vary based on the users’ age, gender origin, income, educational attainment, and on whether they live in urban or rural areas [19, 36, 37]. Clearly, Facebook users do not represent the real population, and thus to reduce the impact of the Facebook usage selection bias we introduced the penetration rate as a variable in our models. A second limitation is that the Facebook estimates are accessed in an opportunistic manner. Facebook may change at any time the conditions for accessing the data, it does not disclose the detailed criteria for classifying its users e.g. as a “frequent travellers”, and the classification criteria may change at any time without prior notice [19, 37].

A third limitation, but also a data protection safeguard, is that we have access to anonymized, aggregated and rounded data with a confidentiality threshold of 1000 users. On the one hand, due to the aggregated form of the data, we were not able to apply a more robust bias correction methodology, and due to the 1000 users confidentiality threshold, we did not obtain estimates about age-gender-residence-previous residence groups with less than 1000 users. On the other hand, the aggregated form of the data and the 1000 users confidentiality threshold guarantees that the re-identification of individuals is highly unlikely. Still, the high confidentiality threshold cannot eliminate the risk of exposing data about the location and the behaviour of large vulnerable migrant groups (e.g. displaced populations) when data are collected at a very detailed spatial-temporal resolution. Thus, as also concluded by Rama et al. [37], the use of Facebook audience estimates should be done with caution, especially in high-risk settings, for example in or near conflict zones.

Based on the literature reduced geographical mobility is associated with increased risk of social exclusion [1], reduced psychological well-being [2] and lower-income [3, 4]. When travelling is limited and devoted mostly to compulsory places, the whole experience of space becomes ruled by the sign of necessity, a space of survival rather than a space of belonging [38]. The importance of this study is to offer a novel possibility to build indicators of migrants’ well-being by measuring their geographical mobility. While such indicators can be constructed for specific groups of migrants, it becomes extremely challenging to have a more comprehensive and systematic overview at a global level. Facebook, despite its limitations, offers unprecedented possibilities to generate new statistics on the sociodemographic and behavioural characteristics of the migrants’ population broken down by country of residence, country of origin age and gender. In our study, by analyzing the different mobility patterns of migrants groups, we were able to show how mobility inequalities in the countries of previous residence of Facebook users are being perpetuated in the new countries of residence, a fact that can introduce structural barriers in the smooth integration of migrants, for example of women from countries with conservative gender norms in western societies. However, in order to provide more solid evidence on whether our findings are also valid for the general population, collaboration with Facebook is required to better understand how the data is being produced and pre-processed.

## Supporting information

S1 Table List of countries of previous residence for which Facebook provides audience estimates. (DOCX)
