# Supplementary material for: Explaining the travelling behaviour of migrants using Facebook audience estimates
Source: PLoS One. 2020 Sep 11;15(9):e0238947. doi: 10.1371/journal.pone.0238947 (PMC7485891; doi:10.1371/journal.pone.0238947)
Supplement: S1 Table — (DOCX) [file pone.0238947.s001.docx]

**S1 Table.** List of countries of previous residence for which Facebook provides audience estimates.

| 1 | Algeria | 31 | Hungary | 61 | Poland |
| --- | --- | --- | --- | --- | --- |
| 2 | Argentina | 32 | India | 62 | Portugal |
| 3 | Australia | 33 | Indonesia | 63 | Puerto Rico |
| 4 | Austria | 34 | Ireland | 64 | Qatar |
| 5 | Bangladesh | 35 | Israel | 65 | Romania |
| 6 | Belgium | 36 | Italy | 66 | Russia |
| 7 | Brazil | 37 | Ivory Coast | 67 | Rwanda |
| 8 | Cameroon | 38 | Jamaica | 68 | Senegal |
| 9 | Canada | 39 | Japan | 69 | Serbia |
| 10 | Chile | 40 | Jordan | 70 | Sierra Leone |
| 11 | China | 41 | Kenya | 71 | Singapore |
| 12 | Colombia | 42 | KSA | 72 | Slovakia |
| 13 | Congo DRC | 43 | Kuwait | 73 | Slovenia |
| 14 | Cuba | 44 | Latvia | 74 | South Africa |
| 15 | Cyprus | 45 | Lebanon | 75 | South Korea |
| 16 | Czech Republic | 46 | Lithuania | 76 | Spain |
| 17 | Denmark | 47 | Luxembourg | 77 | Sri Lanka |
| 18 | Dominican Republic | 48 | Malaysia | 78 | Sweden |
| 19 | El Salvador | 49 | Malta | 79 | Switzerland |
| 20 | Estonia | 50 | Mexico | 80 | Tanzania |
| 21 | Ethiopia | 51 | Monaco | 81 | Thailand |
| 22 | Finland | 52 | Morocco | 82 | UAE |
| 23 | France | 53 | Nepal | 83 | Uganda |
| 24 | Germany | 54 | Netherlands | 84 | United Kingdom |
| 25 | Ghana | 55 | New Zealand | 85 | United States |
| 26 | Greece | 56 | Nicaragua | 86 | Venezuela |
| 27 | Guatemala | 57 | Nigeria | 87 | Vietnam |
| 28 | Haiti | 58 | Norway | 88 | Zambia |
| 29 | Honduras | 59 | Peru | 89 | Zimbabwe |
| 30 | Hong Kong | 60 | Philippines |  |  |
